# Influence of Shelter and Hibernation on the 24-Hour Behavioral Rhythms of Male Dybowski’s Frog (*Rana dybowskii*) Across Age Groups

**DOI:** 10.3390/ani16060978

**Published:** 2026-03-20

**Authors:** Yingdong Li, Meizhang Wang, Haoyu Ji, Xian Zhang, Baolong Shan

**Affiliations:** College of Animal Science and Veterinary Medicine, Shenyang Agricultural University, Dongling Road 120, Shenyang 110866, China

**Keywords:** *Rana dybowskii*, hibernation, behavioral patterns, shelter, circadian rhythms

## Abstract

Understanding how environmental factors such as shelter availability and hibernation influence amphibian behavior is critical for improving conservation and captive management. In this study, we examined the effects of shelter and hibernation on 24 h behavioral patterns of male Dybowski’s frogs (*Rana dybowskii*) across different age groups. Twenty adult and twenty juvenile frogs were observed during pre- and post-hibernation periods under controlled laboratory conditions using continuous video monitoring. Both adults and juveniles showed significantly increased resting behavior when provided with shelter, with adults resting more than 70% of the time and juveniles exceeding 80%. Hibernation induced clear changes in circadian rhythms, including shifts in peak activity times, and vocalizations increased post-hibernation in both age groups, reflecting physiological adjustments associated with reproductive activation. Juveniles displayed more pronounced activity rhythms and greater sensitivity to shelter availability, indicating age-specific differences in environmental adaptability. These findings demonstrate the importance of shelter in shaping behavioral patterns and highlight that hibernation leads to temporary but marked alterations in daily activity cycles.

## Simple Summary

This study examines how shelter and hibernation affect the daily behavior of Dybowski’s frogs. We observed both adult and juvenile frogs in a controlled setting before and after hibernation. Our results show that frogs rest more when provided with shelter, with juveniles resting more than adults. After hibernation, frogs became more active and vocal, possibly due to reproductive readiness. Juvenile frogs exhibited a stronger behavioral response to the presence of shelter, indicating a higher dependency on environmental structures for safety and comfort compared to adults. These findings highlight the importance of shelter in conserving energy and helping frogs adapt to environmental changes. Protecting habitats with adequate cover is crucial for the survival of frog populations, especially as climate change impacts their ecosystems. This research helps us understand frog behavior better and supports conservation efforts to protect these amphibians and their habitats.

## 1. Introduction

Determining the behavioural rhythms of an animal is crucial for understanding how it interacts with its environment and how these rhythms influence its survival, reproduction, and ecological role [1]. In amphibians, these rhythms are particularly important because they rely on external environmental conditions such as temperature and light cycles for physiological regulation [2,3]. The ectothermic nature, permeable skin, and life history of amphibians make them highly sensitive to environmental changes, affecting their daily and seasonal activity patterns [4]. Studying these behavioral rhythms provides insights into how amphibians adapt to fluctuating conditions, aids in conservation efforts, and improves the design of ecological surveys [5]. Understanding these rhythms is particularly relevant in species that experience marked seasonal transitions, such as those living in temperate zones, where wintering strategies significantly alter their activity cycles [6].

Hibernation is a critical survival strategy for amphibians, allowing them to endure extreme environmental stress, such as cold temperatures and food scarcity [7]. Before entering hibernation, amphibians undergo significant physiological changes, including a decrease in metabolic rate (e.g., *Rana chensinensis* accumulating liver glycogen reserves up to 40% body weight [8]) and hormonal regulation alterations, preparing them for dormancy (e.g., plasma corticosterone surge in *Bufo gargarizans* triggering hibernation preparation) [6,8]. In particular, *Rana dybowskii* exhibits a specialized underwater hibernation strategy in lotic environments, involving significant metabolic suppression and shifts in microbiota to survive long-term hypoxia and cold [9]. Behavioral adaptations are equally important during this transition, as they help these organisms optimize their energy reserves and minimize predation risks [10]. Numerous studies have demonstrated that various behaviors in amphibians, such as calling and locomotion, exhibit distinct circadian and seasonal rhythms [11,12]. These behavioral patterns are crucial for understanding how amphibians interact with their environment and optimizing their survival strategies [5]. However, the existing studies on related species, like *Rana temporaria*, have shown significant reductions in locomotor activity and shifts to nocturnal patterns during pre-hibernation [13]. For *R. dybowskii*, previous research has primarily focused on physiological and microbial changes, leaving a gap in our understanding of its behavioral rhythmic adjustments [11].

The quantification of amphibian behavior relies on diverse recognition approaches, ranging from automated tracking to manual video decoding [14]. While automated systems offer high efficiency for active organisms, their reliability often decreases in aquatic or hibernation studies. Factors such as water refraction and the extremely low-intensity movements of dormant individuals present significant challenges for machine learning algorithms, which may struggle to distinguish quiescent frogs from their environment [15]. Consequently, high-resolution video recording combined with meticulous manual analysis remains essential for hibernating species. This approach ensures the detection of subtle behavioral shifts and low-frequency events, providing the necessary granularity to accurately assess the rhythmic plasticity of amphibian under controlled conditions.

Dybowski’s frog (*Rana dybowskii*), native to northeastern China, is predominantly found in the Changbai and Xiao Xinganling mountains and is highly adapted to cold environments [16]. It hibernates from October to February and breeds from February to June [9]. By feeding on small invertebrates, it plays a crucial ecological role in controlling insect populations [17]. Its oviduct, Oviductus ranae, is a valuable traditional Chinese medicinal material with immunomodulatory, antioxidant, and estrogen-like properties [18]. Owing to its high market demand and classification as a red-listed vulnerable species (IUCN category: Near Threatened), wild populations of *R. dybowskii* are facing a severe decline and habitat fragmentation due to overexploitation and environmental changes [19,20,21]. Improved breeding and propagation practices are urgently needed to support the conservation of *R. dybowskii* populations and the maintenance of their ecological roles within the local ecosystem [22,23]. This study focused on male Dybowski’s frogs because their behavioral patterns during the breeding season are more pronounced and consistent, especially due to mate competition and territorial displays [11]. Unlike females, whose behaviors may vary depending on reproductive status (e.g., ovulation or egg-laying), males exhibit more stable activity rhythms, providing a clearer baseline for analyzing post-hibernation behavioral changes [24]. However, future work including females is needed to clarify potential sex-specific behavioral adaptations. The aim of this study was to examine how shelter availability and hibernation phase jointly shape circadian behavioral rhythms in Dybowski’s frog across different age classes. We hypothesized that shelter and hibernation phase would modify the temporal organization and amplitude of daily activity rhythms. We further predicted that juveniles would exhibit greater behavioral plasticity in these rhythms than adults, indicating age-specific differences in rhythmic adaptability to environmental factors.

## 2. Materials and Methods

### 2.1. Ethics Statement

Although *R. dybowskii* is listed as “Near Threatened” on the IUCN Red List, in China, it is regarded as an important economic frog species and is neither endangered nor nationally protected. All experiments were performed in accordance with the guidelines for scientific purposes, animal care, and use formulated by the Animal Ethics Committee of Shenyang Agricultural University. All efforts were made to minimize animal suffering as much as possible. Specifically, frogs were housed in spacious tanks with species-appropriate temperature and moisture levels, and stocking density was kept low to reduce stress. Behavioral data were collected via non-invasive video recording, eliminating the need for frequent human handling. Furthermore, the health status of all individuals was monitored daily, and any signs of distress would have resulted in immediate veterinary intervention.

### 2.2. Animals

On 10 October 2023, 100 male adult *Rana dybowskii* (mean weight 24.25 g ± 8.75 g) and 100 male juvenile individuals (mean weight 6.75 g ± 2.75 g) were collected from Dandong Huiying Frog Breeding Farm in Liaoning Province. At this facility, hibernation is carried out under natural conditions: frogs are placed in outdoor enclosures with water pond maintained to mimic natural wintering habitats, allowing them to undergo hibernation without artificial induction. These frogs were transported to the Aquatic Biology Laboratory of Shenyang Agricultural University and housed in large glass tanks (120 × 80 × 50 cm) filled with 10 cm depth of dechlorinated tap water, which was continuously aerated and renewed every 48 h. Each tank was provided with PVC pipes and smooth rocks to serve as shelters and resting sites. The laboratory room was maintained at 20–23 °C with relative humidity 60–70%, a 12 h light/12 h dark cycle provided by LED lamps (500–600 lx), and filtered air circulation. Frogs were fed daily with live blackworms (*Tenebrio* spp.) at 3% of their body weight.

From this initial pool, 12 adults and 12 juveniles were randomly selected for the pre-hibernation behavioral experiments (*n* = 24 total for the pre-hibernation phase), ensuring standardized density in the experimental tanks. The remaining individuals were kept as a backup population to maintain a stable environment in the holding tanks but were not used for video recording (Figure S1).

A second cohort of 100 male adult (mean weight 22.78 g ± 6.37 g) and 100 male juvenile frogs (mean weight 7.66 g ± 3.21 g) was collected from the same site on 10 May 2024 following natural emergence from hibernation. These individuals underwent identical acclimatization protocols to ensure consistency between pre- and post-hibernation experimental groups.

### 2.3. Experimental Setup and Conditions

The experiment followed a fully crossed factorial design with three fixed factors:

(1) Hibernation phase (pre-hibernation vs. post-hibernation);
(2) Age class (adult vs. juvenile);
(3) Shelter condition (shelter vs. no shelter).

Pre-hibernation experiments were conducted in October 2024, and post-hibernation experiments were conducted in April 2025. For each hibernation phase, twelve glass tanks (120 × 80 × 50 cm) were used. Frogs were housed by age class, with six tanks assigned to adults and six to juveniles. Within each age class, tanks were evenly divided between shelter and no-shelter treatments (*n* = 3 tanks per treatment), with two individuals per tank. In the shelter treatment, five PVC hides (25 × 15 × 10 cm) and five smooth rocks (15 × 10 × 10 cm) were provided to simulate natural microhabitats. No hides or rocks were added in the no-shelter treatment. Environmental conditions were standardized across all tanks and phases, with temperatures maintained at 20–23 °C, relative humidity at 60–70%, and a 12 h:12 h light–dark photoperiod (500 lux during the light phase) provided by LED lighting.

Each tank was equipped with a fixed infrared camera (HIKVISION, 3T66WDV3, Hangzhou, China) mounted on one side of the tank to ensure an unobstructed view of the entire aquatic and terrestrial area. The host utilized for video recording was a HikVision digital video recorder with the specifications of DS-8104HF-ST (Hangzhou Hikvision Digital Technology Co., Ltd., Hangzhou, China). The output resolution was 1280 × 1024/60 Hz with a frame rate of 25 (PAL)/30(NTSC). Video recordings were manually analyzed to quantify a suite of behavioral metrics, including the proportion of time spent, frequency and duration on different behavior. These behavioral descriptors were selected based on established protocols [14].

### 2.4. Behavioral Observation and Analysis Methods

Behavioral data were obtained from continuous 24 h video recordings over two separate 10-day periods, corresponding to pre-hibernation and post-hibernation phases. Observations combined scan sampling and focal animal observations [25]. In scan sampling, activity states (active, inactive, sheltering) were recorded for all individuals at fixed 5 min intervals to quantify group-level activity rhythms [26,27]. For focal observations, one individual per tank was selected based on optimal visibility (closest to the camera) and observed continuously for 15 min, during which locomotor events, shelter use, and posture were recorded. A new focal individual was then selected, and this procedure was repeated twice per hour across the 24 h period. Each individual was cumulatively recorded for 12 h per day (i.e., 15 min + 15 min of observation per hour across 24 h).

Behavioral categories and definitions are summarized in Table 1. For each behavior, total duration per individual per 24 h period was calculated and then averaged across all individuals within the same experimental group. This procedure was applied consistently for both pre- and post-hibernation phases, as well as for the two environmental conditions (with vs. without shelter), ensuring standardized comparisons among treatment groups. To assess reliability, two trained observers independently scored all videos, and the final values for each behavior were calculated as the average of their scores.

### 2.5. Data Statistics

Statistical analysis was performed using IBM SPSS Statistics 27. Behavioral data (duration of each behavior) were analyzed using General Linear Mixed Models (GLMMs) to assess the main effects and interactions of three fixed factors: (1) age class (adult vs. juvenile), (2) hibernation phase (pre-hibernation vs. post-hibernation), and (3) shelter condition (shelter vs. no-shelter). The assumptions for parametric analysis were verified by confirming the normality of standardized residuals (Table S1) and homogeneity of variances (Levene’s test, Table S2), validating the use of the GLMM framework for the primary behavioral metrics (specifically, the three primary behaviors—resting, crawling, and jumping—strictly adhered to the parametric assumptions; for the two secondary active behaviors (calling and swimming) that exhibited non-normality due to zero-inflation, additional cross-validation using Kruskal–Wallis H tests confirmed that the analysis identified significant trends and biological conclusions remained consistent). Post hoc comparisons were conducted using the Bonferroni test to identify specific differences between treatment combinations when significant interactions were detected. Results are presented as mean ± SD, and the significance level was set at *p* < 0.05.

## 3. Results

### 3.1. Daily Allocation of Frog Behavior

**Figure 1:**
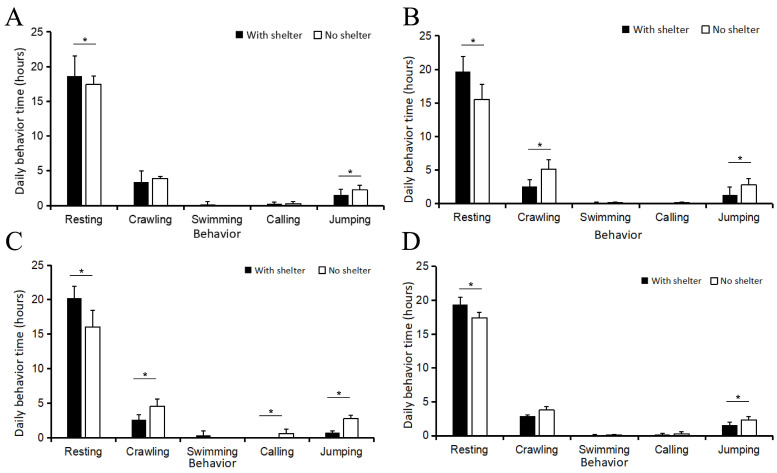
Daily time allocation of behavioral activities in frogs during different periods and age groups: (**A**) adult frogs before hibernation, (**B**) juvenile frogs before hibernation, (**C**) adult frogs after hibernation, and (**D**) juvenile frogs after hibernation. “*” indicate significant differences between time points or groups (*p* < 0.05).

The linear mixed model and behavioral analysis revealed that shelter availability was the most critical factor influencing the frogs’ daily time budget (Table S3). Resting and Crawling represented the two behavioral extremes in terms of time allocation across all groups (Figure 1).

For adult frogs, resting behavior accounted for more than 16 h per day under all conditions, with the shelter group exhibiting significantly longer resting durations than the no-shelter group both before (18.59 vs. 17.42 h) and after (20.18 vs. 15.99 h) hibernation. In contrast, jumping behavior was most pronounced in the no-shelter group after hibernation, reaching 2.71\pm 0.54 h, nearly four times the duration observed in the shelter group (0.68\pm 0.54 h; t = 5.052, *p* < 0.001). Additionally, calling behavior was virtually absent in the shelter group post-hibernation (−0.033 h, approximating zero), while the no-shelter group showed a slight but notable increase to 0.54\pm 0.54 h (Figure 1A,C).

Juveniles exhibited the highest levels of active movement (crawling) among all test subjects. Specifically, before hibernation, crawling in the no-shelter group reached a maximum of 5.12\pm 0.54 h per day (Figure 1B), significantly exceeding the 2.48\pm 0.54 h recorded in the shelter group (t = 9.545, *p* < 0.001). Similar to adults, resting reached its maximum in the shelter-provided environment, peaking at 19.62\pm 0.54 h during the pre-hibernation phase (Figure 1D).

### 3.2. Circadian Behavioral Rhythms of Adult Frogs

**Figure 2:**
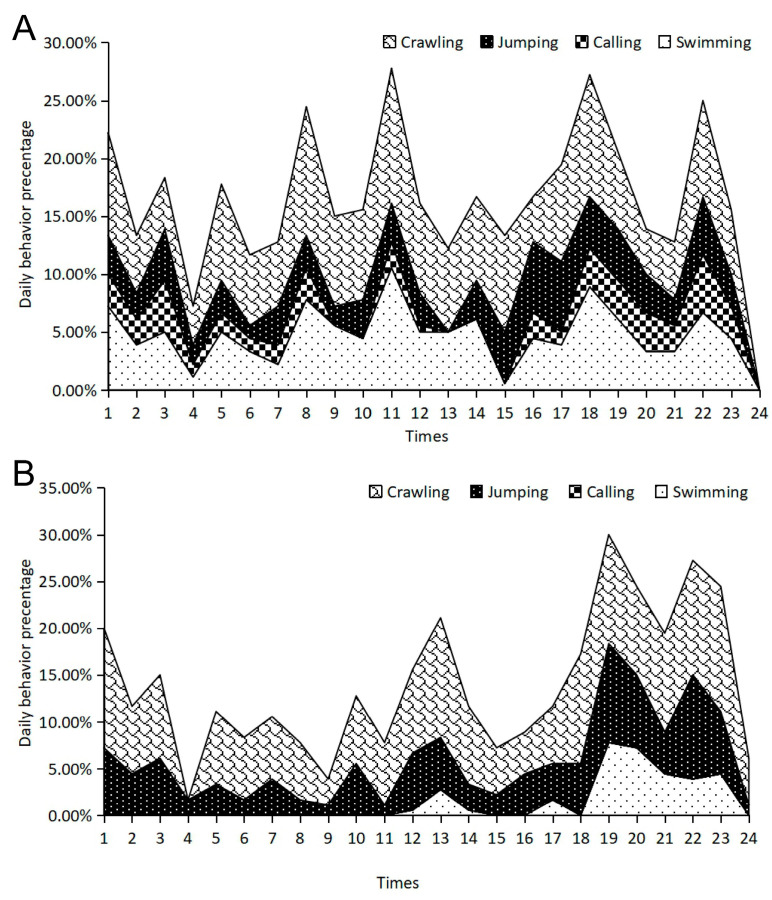
Circadian behavioral rhythms of adult frogs during pre-hibernation with shelter (**A**) and without shelter (**B**).

During the pre-hibernation period, adult frogs in sheltered environments exhibited a highly structured circadian rhythm characterized by multiple activity peaks (Figure 2A). The linear mixed model confirmed that the primary activity peak occurred at 11:00, where the cumulative active behaviors reached their maximum percentage. This was driven by a peak in crawling (t = 187.11, *p* < 0.001) and swimming (t = 158.39, *p* < 0.001), suggesting high mobility during the late morning (Table S4). A secondary major peak was observed at 18:00, coinciding with the onset of the evening phase. At this time, crawling (t = 158.39, *p* < 0.001) and swimming remained the dominant active behaviors. Additional rhythmic peaks were confirmed at 08:00 and 22:00, with the latter (22:00) being particularly notable for the occurrence of calling behavior, which showed a significant positive coefficient, and a small increase in jumping activity (t = 14.49, *p* < 0.001).

Frogs without shelter displayed markedly different circadian patterns compared to the sheltered group, characterized by a significant suppression of daytime activity and a shift toward nocturnal movement (Figure 2B). Specifically, daytime crawling and swimming were substantially reduced, with crawling showing significant negative or lower coefficients during the typical morning peak hours (e.g., 04:00: t = −35.79, *p* < 0.001). Instead of the robust multi-modal rhythm seen in sheltered frogs, the no-shelter group exhibited more concentrated activity peaks in the evening. The most prominent activity occurred at 19:00, where swimming behavior reached its daily maximum (t = 107.70, *p* < 0.001), and at 22:00, marked by a peak in crawling (t = 125.43, *p* < 0.001) and a slight increase in jumping (t = 18.06, *p* < 0.001). Notably, calling behavior was completely absent throughout the 24 h period (Table S5).

**Figure 3:**
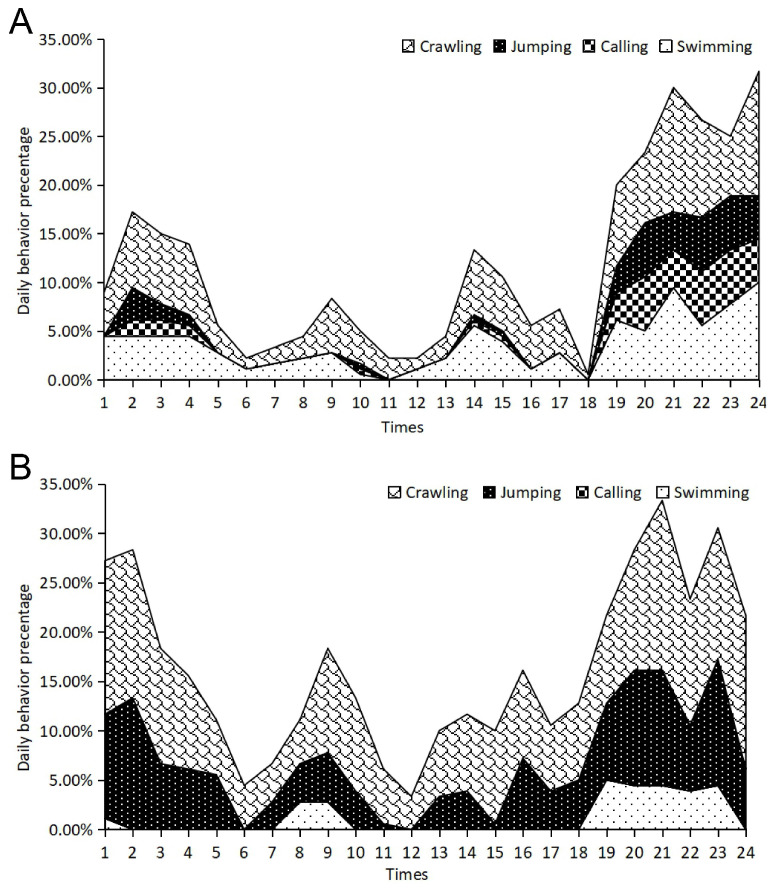
Circadian behavioral rhythms of adult frogs during post-hibernation with shelter (**A**) and without shelter (**B**).

During the post-hibernation period, sheltered frogs displayed a similar activity peak at 24:00, consistent with their pre-hibernation nocturnal behavior (Figure 3A). This peak was statistically driven by crawling behavior (t = 134.93, *p* < 0.001), while a significant secondary peak in swimming occurred earlier at 21:00 (t = 44.80, *p* < 0.001) (Table S6). However, there was a notable shift in the morning activity peak, moving to 06:00, indicating a possible post-hibernation adaptation. The no-shelter group exhibited severely flattened and fragmented rhythms (Figure 3B). While crawling remained their most frequent activity (e.g., at 21:00: t = 37.01, *p* < 0.001), the overall activity intensity was markedly lower than that of the sheltered group (Table S7). Furthermore, calling behavior remained entirely suppressed throughout the 24 h cycle in the absence of shelter (t = −12.79, *p* < 0.001), indicating that environmental stress continues to inhibit social signaling even after hibernation.

### 3.3. Circadian Behavioral Rhythms of Juvenile Frogs

**Figure 4:**
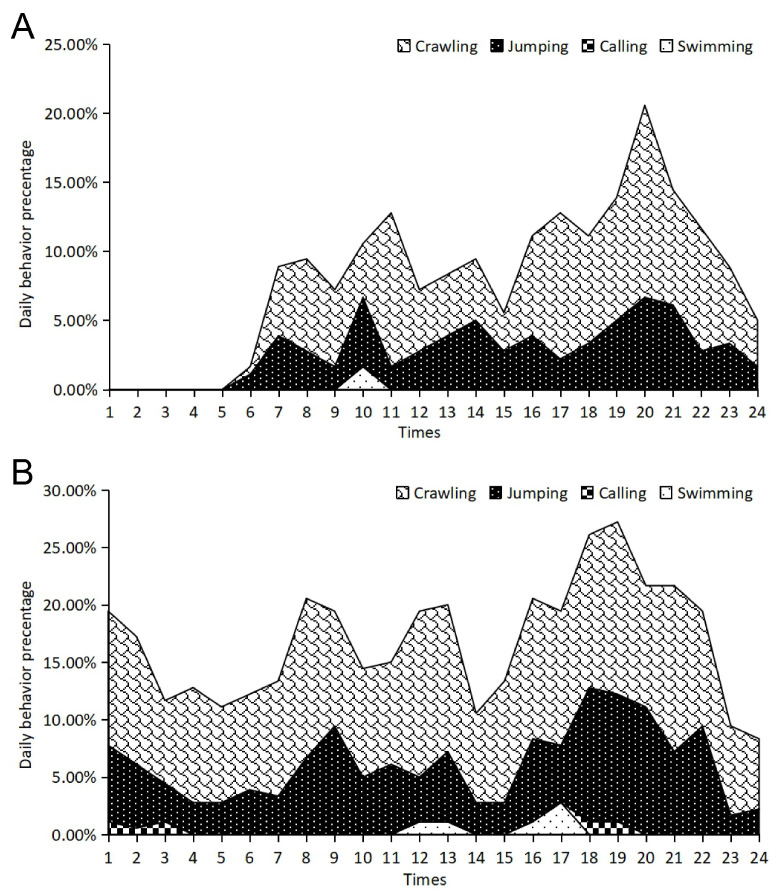
Circadian behavioral rhythms of juvenile frogs during pre-hibernation with shelter (**A**) and without shelter (**B**).

Similar to adult frogs, juvenile frogs in covered environments exhibited peaks in activity at 18:00 and 04:00, indicating significant evening activity (Figure 4A). Statistical analysis confirmed that the highest activity peak occurred at 20:00, predominantly driven by crawling (t = 540.78, *p* < 0.001) and jumping behaviors (t = 259.68, *p* < 0.001) (Table S8). In contrast to adult frogs, sheltered juveniles showed minimal activity from 01:00 to 05:00, where most active behaviors were near zero. In the absence of shelter, juvenile frogs displayed a significantly altered and more fragmented behavioral rhythm (Figure 4B). The primary evening peak shifted earlier to 19:00, where crawling reached its maximum coefficient (t = 88.97, *p* < 0.001) (Table S9). The rhythms appear flattened, indicating that the lack of shelter before hibernation disrupts the strength and regularity of behavioral cycles.

**Figure 5:**
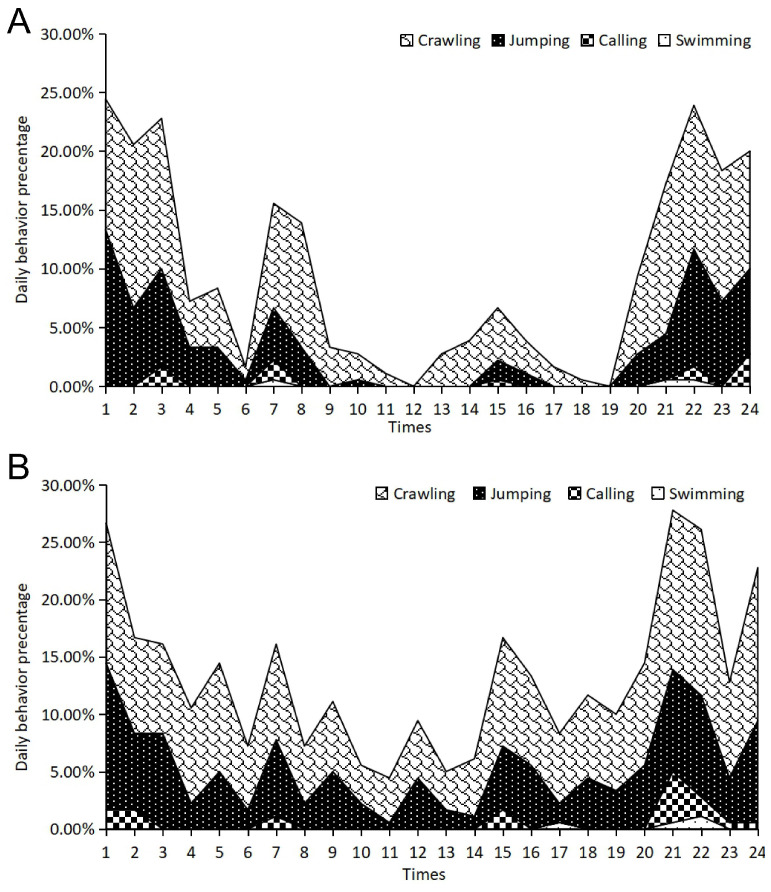
Circadian behavioral rhythms of juvenile frogs during post-hibernation with shelter (**A**) and without shelter (**B**).

After hibernation, sheltered juvenile frogs showed a robust multimodal rhythm, though with notable temporal shifts compared to the pre-hibernation period (Figure 5A). The primary nocturnal peak occurred at 22:00, characterized by a strong convergence of crawling (t = 205.19, *p* < 0.001) and jumping behaviors (t = 160.56, *p* < 0.001) (Table S10). For juvenile frogs without shelter, the post-hibernation rhythms remained fragmented and lacked clear peak-trough transitions (Figure 5B). Although these individuals showed a peak at 21:00–22:00 (Crawling: t = 407.86, *p* < 0.001) (Table S11), their overall activity was distributed more evenly across the 24 h cycle compared to the sheltered group.

## 4. Discussion

Environmental factors consistently influence frog behavior, and studying these behaviors in controlled environments provides valuable insights into how specific environmental conditions shape activity patterns [28]. Behavioral observations focused on crawling, swimming, resting, and calling, which are clearly defined in Table 1. Repeated video analysis allowed observers to reliably identify and record these behaviors, improving both the accuracy and efficiency of data collection. For instance, Luo et al. (2025) employed a similar method in their study of piglets, where distinct behavioral patterns were identified through iterative video review, enabling precise and rapid annotation of complex social behaviors [29]. This research enhances our understanding of *R. dybowskii*’s behavioral adaptations under controlled conditions and provides insights into the species’ activity patterns, locomotor strategies, and use of shelter.

### 4.1. Influence of Shelter on Behavioral Patterns

Our provisional findings indicated that Dybowski frogs prefer environments with adequate shelter to exposed conditions [30]. This preference aligns with observations in other amphibian species, including *Xenopus laevis* and *Agalychnis callidryas*, which also favor sheltered environments, leading to reduced clumping behavior, daytime activity, and aggressive encounters [31]. This is valuable information for captive management, as providing appropriate shelter can improve frog welfare, reduce stress-induced behaviors, and enhance survival and reproduction rates in artificial environments [32]. In sheltered environments, both adult and juvenile frogs demonstrated increased swimming activity and a greater variety of behaviors throughout the day. In contrast, frogs in the uncovered environments exhibited a predominance of active behaviors, such as jumping and crawling, likely as a response to increased predation risk and competition for resources. This is similar to crawfish frogs in post-burn habitats, which are highly visible and thus more vulnerable to predation [33].

### 4.2. Influence of Frog Age on Behavioral Patterns

Most studies have found that frogs exhibit consistent nocturnal diel activity patterns throughout their terrestrial life stages [34]. However, Pacific horned frogs (*Ceratophrys stolzmanni*) display an ontogenetic change in the temporal niche used, with adults being strictly nocturnal and juveniles mainly active during the daytime [35]. In the present study, adult frogs exhibited predominantly resting behavior, especially in covered environments, suggesting an energy conservation strategy. This strategy may enable them to efficiently resume essential activities such as foraging and mating when necessary. These findings are consistent with those of previous studies on amphibians, suggesting that adult frogs confer advantages in energy storage and resilience to environmental fluctuations [8].

Differences in the activity patterns of adult and juvenile frogs reflect distinct survival strategies related to body size [11]. Juvenile frogs use both spatial and temporal tactics to avoid adult frogs despite sharing similar food sources because of their lack of preference for prey size [36]. These behavioral differences, including locomotion and vocalization variations, help reduce dietary overlap and competition between age classes [37]. However, larger adult frogs can conserve energy while maintaining reproductive behaviors, providing them with a selective advantage in resource-limited environments. This contrast suggested that body size affects physical capabilities and influences behavioral responses to environmental cues. This may have significant implications for survival, fitness, and resource partitioning. Future research should investigate how size-dependent behavioral adaptations shape population dynamics and habitat use in response to environmental pressures.

### 4.3. Influence of Hibernation on Behavioral Patterns

Hibernating amphibians undergo a series of specialized ecological and physiological adaptations to survive the harsh conditions associated with hibernation. These adaptations include fluctuations in blood hormone levels, the synthesis of cryoprotective proteins and transporters, the accumulation of glucose in tissues to serve as a cryoprotectant, and a metabolic shift towards anaerobic pathways to sustain vital functions under low oxygen availability [38,39]. These physiological changes directly influence their behavioral patterns, resulting in significant differences in activity and priorities between the pre- and post-hibernation periods [40]. However, environmental requirements during dormancy are highly species-specific. For instance, while *R. dybowskii* demands significantly higher dissolved oxygen than *Rana amurensis* [41], other species demonstrate different environmental sensitivities; for example, the migration of *Rana sakuraii* is triggered by specific air temperature thresholds and photoperiod cues [42], and high-altitude *Rana temporaria* populations exhibit altered metabolic rates to facilitate breeding at lower temperatures [43]. In the present study, hibernation significantly altered activity rhythms in *R. dybowskii*, with adults shifting their morning activity peak from 4:00 AM to 6:00 AM and juveniles delaying their evening peak from 6:00 PM to 7:00 PM. Moreover, the resting time after hibernation was approximately 10% longer than that before hibernation. This may be attributed to the immediate onset of mating in *R. dybowskii* following hibernation, a period devoid of feeding, which necessitates increased rest for energy recovery and gonad development prior to reproduction [44]. This pattern aligns with the capital breeding strategy observed in many temperate anurans, where stored energy reserves are prioritized for reproductive activities immediately after hibernation [25].

Both adult and juvenile *R. dybowskii* showed increased vocalizations after hibernation, indicating physiological and ecological adjustments. This phenomenon is comparable to the circadian and seasonal vocal activity patterns observed in mountain yellow-legged frog (*Rana muscosa*), in which vocalization is closely linked to mating and territorial behaviors [45]. For *R. dybowskii*, such changes in vocal behavior likely indicated a shift in priority from energy conservation during hibernation to reproductive readiness and mate attraction post-hibernation [21]. Behavioral observations showed increased vocalization after hibernation, which may reflect the influence of environmental cues on hormonal regulation, thereby stimulating calling behavior [12,16,19]. These findings underscore the adaptive importance of vocalization in *R. dybowskii*, as it facilitates successful reproduction and signals their ability to thrive under dynamic environmental conditions.

## 5. Conclusions

The findings of this study provide initial observations on the behavioral patterns of *R. dybowskii*, indicating potential effects of shelter conditions and hibernation on amphibian activity. The presence of shelter significantly increased the resting time of adult frogs. After hibernation, both adults and juveniles exhibit increased vocalization. These findings underscore the importance of shelter in modulating behavioral rhythms and highlight the role of hibernation in shaping activity patterns. Future research should expand upon these findings by incorporating longitudinal monitoring over multiple hibernation cycles to assess the stability of these behavioral shifts. Given the sexual dimorphism observed in many ranid frogs, including female individuals in future cohorts is essential to clarify sex-specific adaptive strategies. These findings establish a crucial reference point and explicitly identify key temporal windows and behavioral responses that now necessitate and guide more comprehensive, longitudinal investigations.

## Figures and Tables

**Table 1:** Ethogram of state behaviors for captive *Rana dybowskii*, adapted from work on *Xenopus longipes* [26].

Behavior	Description
Resting	The frog stays mostly underwater, with only its eyes and nostrils above the surface. Its hind legs are bent, and the forelegs are relaxed, partly or fully submerged for support.
Crawling	The frog moves slowly on land by extending its hind legs and forelegs alternately, without jumping, often used when navigating uneven surfaces.
Swimming	The hind legs propel movement through leg contraction, web flipping, paddling, and gliding, while the forelegs assist.
Calling	Male frogs inhale air, expand their abdomen, and then contract it to push air to their throat, causing vibrations in the vocal cords. The vocal sac amplifies the sound.
Jumping	The frog crouches and pushes off forcefully with its hind legs to leap forward.

## Data Availability

The original contributions presented in this study are included in the article/Supplementary Materials. Further inquiries can be directed to the corresponding author.

## Supplementary Materials

The following supporting information can be downloaded at https://www.mdpi.com/article/10.3390/ani16060978/s1, Figure S1: Experimental design and sampling framework for behavioral observations of *Rana dybowskii* before and after hibernation; Table S1: Tests of normality for standardized residuals of all behaiovr; Table S2: Levene’s test of equality of error variances; Table S3: Pairwise comparison results from linear mixed-effects models examining effects of shelter condition, hibernation phase, and age class on behavioral durations; Table S4: Pairwise comparison results from linear mixed-effects models examining effects of daily cycle on behavioral durations with shelter condition, pre-hibernation phase and adult conditions; Table S5:Pairwise comparison results from linear mixed-effects models examining effects of daily cycle on behavioral durations without shelter condition, pre-hibernation phase and adult conditions; Table S6: Pairwise comparison results from linear mixed-effects models examining effects of daily cycle on behavioral durations with shelter condition, post-hibernation phase and adult conditions; Table S7: Pairwise comparison results from linear mixed-effects models examining effects of daily cycle on behavioral durations without shelter condition, post-hibernation phase and adult conditions; Table S8: Pairwise comparison results from linear mixed-effects models examining effects of daily cycle on behavioral durations with shelter condition, pre-hibernation phase and juvenile conditions; Table S9: Pairwise comparison results from linear mixed-effects models examining effects of daily cycle on behavioral durations without shelter condition, pre-hibernation phase and juvenile conditions. Table S10: Pairwise comparison results from linear mixed-effects models examining effects of daily cycle on behavioral durations with shelter condition, post-hibernation phase and juvenile conditions; Table S11: Pairwise comparison results from linear mixed-effects models examining effects of daily cycle on behavioral durations without shelter condition, post-hibernation phase and juvenile conditions.
