# Supplementary figures and images for: Influence of Shelter and Hibernation on the 24-Hour Behavioral Rhythms of Male Dybowski’s Frog (Rana dybowskii) Across Age Groups
Source: Animals (Basel). 2026 Mar 20;16(6):978. doi: 10.3390/ani16060978 (PMC13023323; doi:10.3390/ani16060978)

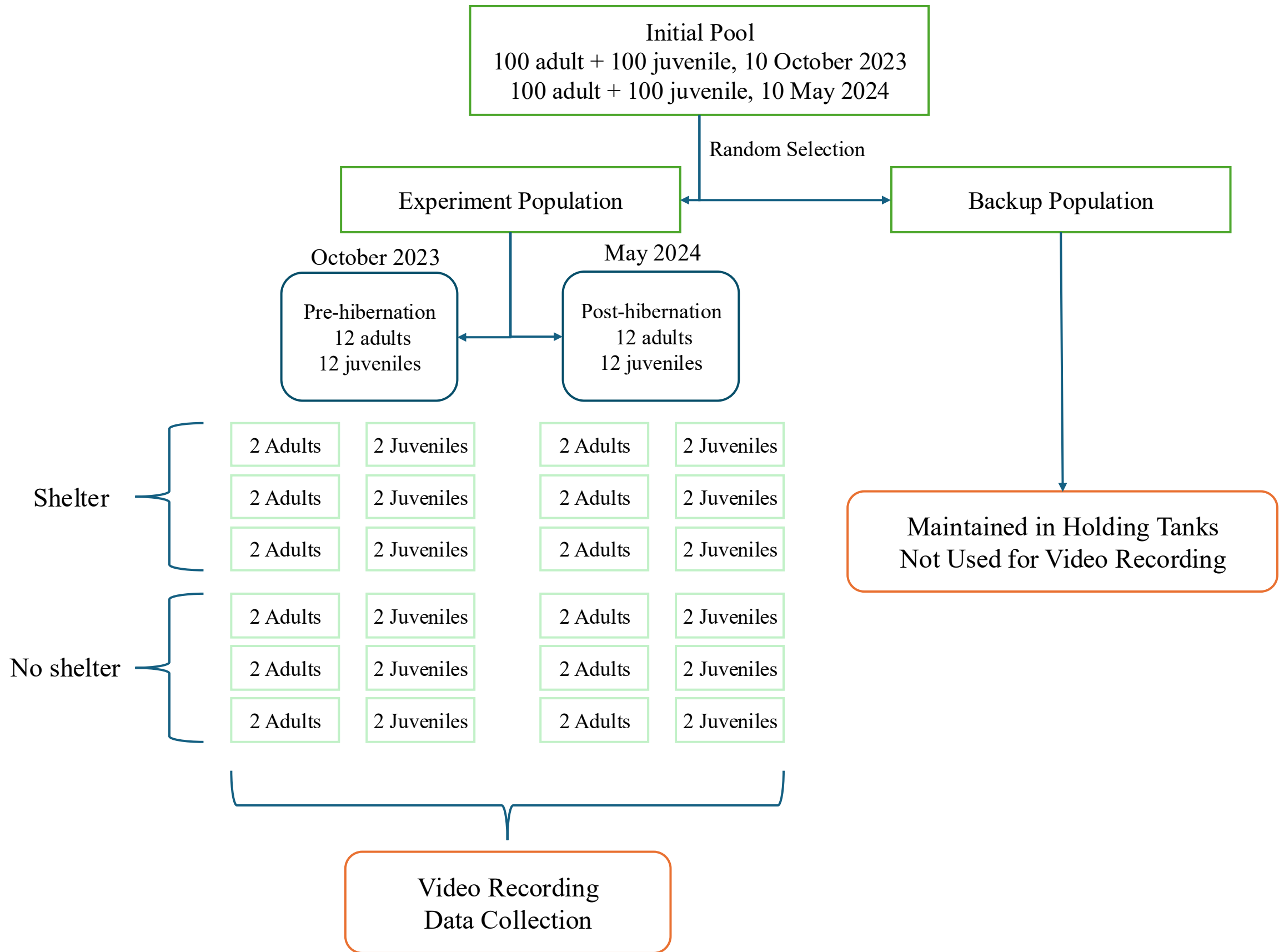

Supplement: Supplementary file 1 [file animals-16-00978-s001.zip › Figure S1.pdf]
